# Attentional Bias for Opioids in Taiwanese Heavy Smokers with Chronic Noncancer Pain

**DOI:** 10.3390/medicina60071107

**Published:** 2024-07-08

**Authors:** Ling-Jun Liu, Edward Meng-Hua Lin, Shao-Lun Tsao, Hsin-Yu Wang, Ming-Chou Ho

**Affiliations:** 1 Department of Anesthesiology, Changhua Christian Hospital, Changhua 500, Taiwan; lingjun.liu1203@gmail.com (L.-J.L.);; 2 Department of Statistics, Tunghai University, Taichung 407, Taiwan; 3 Pharmacy Department, Changhua Christian Hospital, Changhua 500, Taiwan; 4 Department of Psychology, Chung Shan Medical University, Taichung 402, Taiwan

**Keywords:** attentional bias, analgesics, opioid, chronic pain, cigarette smoking

## Abstract

*Background and Objectives:* Attentional bias (AB) for addictive substances is a feature of attention found in individuals with substance misuse or diagnosed with substance use disorders. When AB exists, the attention of the addicted individual may be quickly oriented to cues related to the addictive substance or be maintained on these cues for a longer time. AB toward opioids was found in Western samples of smokers with chronic noncancer pain. The level of AB was dose-responsive. However, similar studies in the Taiwanese population are lacking. This study compared the patterns of AB for opioid analgesics in Taiwanese participants with chronic noncancer pain to that of individuals without pain. This study aimed to investigate if AB toward opioids is presented in Taiwanese heavy smokers who are on long-term opioid therapy for pain control. *Materials and Methods:* Participants were grouped into chronic noncancer pain smokers, chronic pain nonsmokers, and smokers without pain, according to smoking habits and whether or not on long-term opioid therapy for pain control. Each participant completed demographic questionnaires, mood scales, and the opioid-related visual probe task. Differences in AB among the groups were compared using a three-way analysis of covariance controlling for daily cigarette consumption. *Results:* Chronic noncancer pain smokers (*n* = 17) and chronic pain nonsmokers (*n* = 16) displayed more severe levels of depression, anxiety, and pain, compared to smokers without pain (*n* = 28). Only did chronic pain nonsmokers show significant AB for opioid cues that were displayed for a short time. Analysis on reaction time found that smokers without pain consistently responded faster to the tasks. No difference in reaction time was found between the pain groups. *Conclusions:* The current study did not fully replicate findings from studies that were based in Western countries. Formulary availability and regulatory limitations might have affected patient’s perception of prescription opioids in Taiwan. However, chronic pain nonsmokers exhibited initial orientation toward opioid-related cues when daily cigarette consumption was accounted for. According to previous research, this AB for shortly displayed opioid cues can be associated with the expectation of pain relief. The current finding also indicated general psychomotor retardation in individuals who were on long-term use of opioids.

## 1. Introduction

Long-term opioid therapy for chronic pain was related to poorer pain control, worse quality of life, and affective disorders [1]. Several studies have found that smoking further complicates this situation [2,3]. An unsettling relationship has been observed between cigarette smoking and opioid misuse, e.g., [4]. Smokers showed increased rates of being prescribed opioids for pain, even after controlling for the reported level of pain, psychiatric factors, and gender [5]. Smoking is associated with increased opioid use and can worsen pain severity [6,7,8]. There is a higher proportion of smokers among patients with chronic pain than in pain-free people [9,10,11], and the numbers are even higher in opioid users [12]. Patients with chronic pain who take opioid analgesics displayed an increased tendency to be heavy smokers [11]. Furthermore, smoking can be a predictor of future prescription opioid abuse [2].

### 1.1. Smoking and Opioid Misuse in Patients with Pain

A randomized control study reported that the short-term analgesic effect of nicotine is observed only in nonsmokers [13], but animal model and meta-analysis review tended to agree that nicotine can temporarily relieve pain, no matter if the subject (participant) has been previously exposed to nicotine [14,15]. The inconsistencies between these findings could be related to nicotine tolerance. Nicotine has an acute analgesic effect that increases the pain threshold and pain tolerance in both smokers and nonsmokers [15]. However, tolerance to nicotine builds up so fast that the analgesic effect quickly wears off once nicotine is withdrawn. Withdrawal symptoms may stimulate the discomfort experienced by smokers during abstinence [14]. Anxiety accompanying withdrawal from nicotine is typically displayed in mice by the increased proportion of time that mice spent at the perimeter in an open-field task [16]. Human smokers in withdrawal self-reported symptoms, such as anxiety, irritability, difficulty concentrating, gastrointestinal symptoms, nicotine cravings, and insomnia [17]. 

A longitudinal study using smartphones and other handheld devices to track patients’ momentary pain found that, although pain may trigger smoking behaviors in clinical pain patients, the intensity of pain did not abate after smoking [18]. Meanwhile, certain unpleasant body sensations that may in fact be withdrawal symptoms were misinterpreted as the return of pain. Although smoking does not always lead to actual pain reduction in a quantitative measure, the tobacco-seeking behaviors of chronic pain patients remain negatively reinforced by the alleviation of pain-related negative effects [19,20]. Moreover, smokers tended to report more severe pain than nonsmokers in the long run [21,22].

### 1.2. Attentional Bias for Opioids in Chronic Pain

Studies on misuse or addictive behaviors reported attentional bias (AB) for the addicted substance in misusers [23,24]. AB toward substances can be conceptualized as the degree to which one’s attention is captured or held by a stimulus relating to the preferred substances [25,26]. AB is generally assessed with the Stroop test or a visual probe task [27,28,29], and the underlying assumption of these tasks is that certain stimuli, especially those that are feared or favored, can quickly capture an individual’s attention or cause a delay in the shifting of one’s attention. If a person’s attention is systematically drawn to a salient stimulus, a speeded reaction can be expected when a probe replaces the spatial location of the target stimuli (i.e., congruent), compared to a probe that replaces the spatial location of a neutral stimulus (i.e., incongruent). 

Results of cognitive experiments that included patients with parietal lobe deficits explained the delay in reaction time in the incongruent trials. Incongruent trials cause an extension in reaction time, as the participant must disengage from the original location, shift attention, and then re-engage in the new location [30]. Later, Koster et al. (2004) showed that when additional attentional resources were allocated to the stimulus that is most salient to the participants, it would result in an interruption from responding (i.e., difficulty in disengagement) to the task [31]. These earlier studies showed that selective attention toward highly personal relevance stimuli can manifest in cognitive tasks that measure AB. The manipulation of presentation time allowed the researcher to further examine the subcomponents of the attentional process (initial orientation or attentional maintenance) [25]. The shift of attention to the target stimuli presented for less than 200 ms reflected the initial orientation of attention. When the display time was extended, for example, to 2000 ms, attention was able to shift several times between the target and the matched picture. In the scenario with a longer display time, AB for the target indicated the maintained attention [32,33,34]. 

Although previous studies have observed that a large proportion of patients with chronic pain tended to also be heavy smokers [10,12], and cross-tolerance between nicotine and opioids is warned by researchers [35], whether or not smoking habits increase AB for opioids in smokers who are prescribed opioids for pain control is scarcely investigated. 

Only a few studies on AB for opioids in pain patients have been conducted, e.g., [24,34]. The attention of pain patients with opioid dependence exhibited a more rapid orientation toward opioid cues in a visual probe task [24]. Another study included data from patients who had undergone 3 months of behavioral treatments, such as mindfulness training and support group attendance [34]. Results showed that AB scores and cue-elicited craving could predict future risk of opioid misuse [34]. Relative to non-misusers, opioid misusers displayed a tendency to attend to opioid cues above other cues [36]. 

Moreover, there is very little research adopting the visual probe method to study the same issue in the Asian pain population. There has only been one such study listed in a systematic review [37]. Accessibility and attitudes toward opioids can both be factors influencing the use of opioids in Asian countries [38,39]. Compared to Western countries, opioid consumption for pain control remains low in most Asian countries [40]. Taking cultural adaptation into consideration [41], the current study investigates AB for opioids in Taiwanese chronic noncancer pain smokers relative to chronic pain nonsmokers and smokers without pain.

## 2. Materials and Methods

### 2.1. Participants

All participants were recruited from Changhua Christian Hospital; pain participants were recruited from the pain clinic at the hospital, and nonclinical participants were recruited from passersby at the hospital. The study recruited 61 participants, who were further classified into three groups according to their opioid use and smoking statuses: chronic noncancer pain smokers (*n* = 17), chronic pain nonsmokers (*n* = 16), and smokers without pain (*n* = 28). Chronic pain participants had severe noncancer pain for at least 6 months [11] and had been prescribed strong opioids as the main analgesic for pain relief for more than 3 months [24]. Before the recruitment, the pain patients had been registered in the Taiwan Food and Drug Administration (FDA) database for their long-term use of strong opioid analgesics for the management of noncancer pain. Abiding by the guidelines of long-term prescription of narcotic analgesics for noncancer pain patients published by the Taiwan FDA, these patients were interviewed by both a pain physician and a psychiatrist before their registration. For each, the physician determined that the level of pain was sufficiently severe for the prescription of strong opioid analgesics, and the psychiatrist attested that the patients were taking strong opioids for pain management, not for leisure use. Patients in this study may be prescribed the following strong opioid analgesics: morphine, oxycodone, hydromorphone, and fentanyl. Patients with a cancer diagnosis were excluded. 

As in prior studies, the participants self-reported their smoking statuses [2,21]. A smoker was defined as a person who smoked at least 10 cigarettes per day, and this habit lasted for at least 12 months over the previous year. E-cigarette smokers were excluded. To avoid ambiguity in defining nonsmokers, chronic pain nonsmokers in this study included only pain participants who had never smoked or had quit smoking for at least three years. Participants with a compromised ability to understand the questionnaires, and visual probe task instructions were excluded. 

The study was approved by the Institutional Review Board of Changhua Christian Hospital (reference number 171214). Written informed consents were obtained from all participants. For compensation, each participant was given 300 NT Dollars.

### 2.2. Measures

#### 2.2.1. Demographic Questionnaire

Information regarding age, level of education, and smoking status (i.e., years of smoking, amount of daily cigarette consumption, and dependence) was recorded.

#### 2.2.2. Center for Epidemiologic Studies Depression Scale 

This 20-item scale was used to evaluate the severity of depressive symptoms [42,43]. Internal consistency was reported to range between 0.85 and 0.90, and test–retest reliability over 2 to 8 weeks ranged from 0.51 to 0.67. The response options for each item were zero to three, with zero meaning rarely or never, and three indicating almost all the time. Total scores above 16 can indicate signs of depression [44].

#### 2.2.3. Beck Anxiety Inventory 

This 21-item inventory was developed to assess anxiety [45]. The Chinese version of the Beck Anxiety Inventory has been shown to be a valid and reliable measure of the assessment of anxiety (Cronbach’s alpha = 0.95) [46]. The response options for each item were zero to three, and the total score ranged from 0 to 63. A previous study suggested that a cutoff point over 14 or under 13 is best for differentiating between anxious and non-anxious individuals [46].

#### 2.2.4. Pain Score

The participants provided a self-rated score to indicate the average level of pain (0 to 10) experienced over the majority of the time within a week prior to participation. 

#### 2.2.5. Fagerström Test for Nicotine Dependence 

This six-item self-report questionnaire was used to measure the participants’ dependence on nicotine. It possesses acceptable internal consistency (Cronbach’s alpha = 0.61) [47]. Test–retest reliability was reported to range from 0.65 to 0.91 [48]. Total scores can range from 0 to 10, with scores over 5 indicating high levels of physical substance dependence.

#### 2.2.6. Evaluation of Opioid-Use Behaviors

A pharmacist calculated the patients’ average daily morphine milligram equivalent according to their prescriptions from the most recent five clinical visits before the beginning of participation. 

#### 2.2.7. Visual Probe Task

A visual probe task was programmed and presented using E-prime 2.0 [49] on a laptop with a diagonal screen measure of 15 inches. The screen was positioned 45 cm from the participants, who responded using a wireless keyboard that was positioned within an accessible distance. This task is commonly used to examine AB for addictive substances [25]. The opioid-related images included pictures of morphine tablets, capsules, bottles of morphine, fentanyl patches, and the outer case of pills. These pictures were pre-rated by 2 pain physicians and 13 pharmacists at this hospital (7 males and 8 females, mean age = 40 ± 8.35, mean work experience = 12.53 ± 6.68 years), who encountered these medications frequently, to ensure that the pictures did accurately represent the intended drugs commonly prescribed herein. These clinicians also completed one round of the visual probe task to ensure that simple familiarity with the drugs would not confound the results. The mean AB score found in these clinicians was 0.96 (*SD* = 23.47), which did not significantly differ from zero (*t* = 0.16, *p* = 0.876), meaning that these clinicians did not show AB for the opioid-related images. In other words, familiarity with the drugs would not induce AB for the substances. 

**Figure 1:**
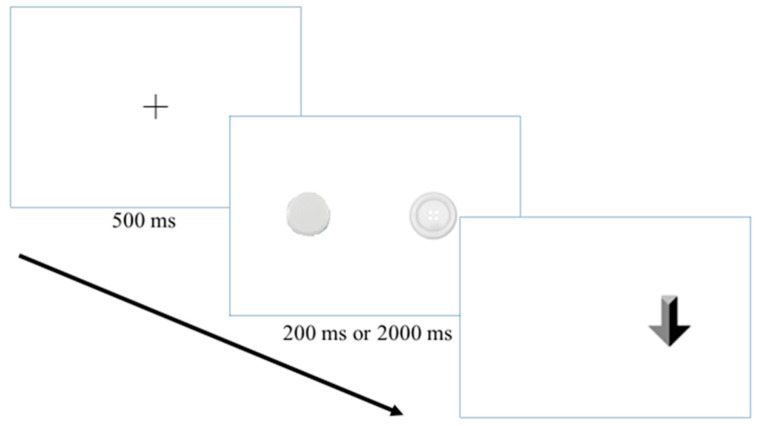
Schematic illustration of an incongruent trail in the visual probe task.

In the visual probe task, participants were presented with two simultaneously appearing visual stimuli that had comparable features. Each trial began with a fixation cross appearing for 500 ms before the onset of the photos, and then the paired photos would appear for 200 or 2000 ms. Afterward, a probe (arrow) situated 5-degree visual angle from the central fixation point that indicated either the up or down direction would appear on either side of the screen, replacing the target (congruent) or the matched neutral picture (incongruent). The participants were instructed to indicate as quickly as possible the direction in which the probe was pointing as soon as it appeared (Figure 1). Figure 1 is an illustration of an incongruent trial. The long arrow on the bottom left indicates the order of appearance of the scenes. A fixation cross was replaced by a pair of target images with a matched neutral image. The matched pair would be displayed for 200 or 2000 ms. Afterward, a thick arrow (i.e., the probe) replaced the location of the matched neutral picture (incongruent). The participant should quickly respond to the task by pressing the arrow key on the keyboard that indicates the direction in which the thick arrow was pointing. 

Facilitated reaction time for the probe appearing in the congruent location of the target reflected the allocation of attention toward the target stimulus. The manipulation of the stimulus display duration enabled the investigation of different stages of attentional processing [28]. In congruent trials, the speeded reaction time for a target displayed for 200 ms reflected an initial orientation toward the stimuli [41,50], but the presentation time of 2000 ms allowed the eyes to saccade, so the AB might disappear in longer presentation trials [41]. 

Different latencies in the time that the participants reacted to both scenarios were calculated to indicate a shift in attention [26]. AB scores were calculated as the mean differences obtained by subtracting the reaction time in congruent trials from that in incongruent trials. A positive score indicated AB toward the target [24].

#### 2.2.8. Visual Probe Task Apparatus 

Before the formal trials, 16 practice trials were performed. Each formal cycle consisted of 120 trials with 80 critical trials presented for either 200 or 2000 ms (40 each), and 40 filler trials were presented for either duration. The target trials contained a color photograph of the target (opioid-related), and a matched neutral photo (e.g., morphine tablets versus buttons of the same color), both 7.6-degree visual angle in length and 5.4-degree visual angle in width. The filler trials contained only two neutral photos, which were matched according to complexity, color, and figure–background relationship. Each of the 10 pairs of target–matched photos was presented randomly for 200 or 2000 ms with equal probability. The target and matched photos were 3.0-degree visual angles apart (edge to edge) when simultaneously presented on the monitor and appeared at the left or right of the screen with equal probability. The probe replaced the target or matched photo with equal probability. An additional 20 pairs of non-target stimuli photographs were used as stimuli in the filler trials. The reaction times in the filler trials were excluded from the analysis.

### 2.3. Procedures

Each participant completed the demographic questionnaire, self-rated pain, Center for Epidemiologic Studies Depression Scale, Beck Anxiety Inventory, Fagerström Test for Nicotine Dependence, and the visual probe task.

### 2.4. Data Analysis

The data were analyzed using R (version 4.3.1) [51]. Differences between groups were analyzed with univariate analysis of variance (or a *t*-test, where appropriate). Analysis of covariance (ANCOVA) controlling for years of smoking and daily cigarette consumption was used to compare the AB scores among the three groups. To investigate the differences in reaction time between the groups, a 3-way (groups: chronic noncancer pain smokers, chronic pain nonsmokers, and smokers without pain) × 2 (display time: 200 and 2000 ms) × 2 (probe location: congruent and incongruent) ANCOVA controlling for years of smoking or daily cigarette consumption was performed. The investigation of reaction time is meaningful only when the result of AB is significant. Therefore, whether the ANCOVA on reaction time controlling for years of smoking or daily cigarette consumption should be conducted will be dependent on the significance of the ANCOVA on AB.

To determine the minimum sample size required to test the study hypothesis, an a priori power analysis was conducted using G*Power version 3.1.9.7 [52]. The required sample size to achieve power = 0.8 for detecting a medium effect, at α = 0.05, was N = 84. 

### 2.5. Data Availability

This study reports the following information: how the sample size was determined, all data exclusions, all manipulations, and all measures in the study, and we follow Journal Article Reporting Standards [53]. Raw data and analysis code for the ANCOVA are available as Supplementary Materials. Data were analyzed using R, version 4.3.1 [51], packages rstatix version 0.7.2 [54], readxl [55], ggpubr version 0.6.0 [56], and ggplot2 [57]. Neither the study design nor analytical plans were pre-registered.

## 3. Results

### 3.1. Demographic Characteristics

Daily cigarette consumption, years of smoking, and total scores on the Fagerström Test for Nicotine Dependence were similar between the chronic noncancer pain smokers and smokers without pain groups (Table 1). Post hoc analysis using Scheffe’s method on other variables among the three groups showed that chronic noncancer pain smokers and chronic pain nonsmokers displayed comparable levels of depression, anxiety, and pain (all *p*s > 0.649). These groups also had greater severity in all three measures compared to smokers without pain (all *p*s < 0.01) (Table 1). The levels of morphine milligram equivalent were comparable between the chronic noncancer pain smokers and chronic pain nonsmokers.

### 3.2. Attentional Bias and Reaction Time

Formal trials in the visual probe task were analyzed. The participants performed these tasks with an error rate of less than 10%. Incorrect responses, correct responses with reaction times shorter than 200 ms, and reaction times over three standard deviations above the mean reaction time were removed. A total of 1.91% of the trials were eliminated from analyses. Details of reaction time and bias scores are presented in Table 2. Results of ANCOVA on AB scores and reaction times will be reported in later paragraphs. The power of the actual sample size (*n* = 61) included in this study was 0.63. However, according to the rules of thumb for determining the sample size, it is acceptable when the sample size for each cell is more than 7 in tests measuring group differences [58]. Therefore, the current result is statistically acceptable.

#### 3.2.1. Analysis on AB Scores

**Figure 2:**
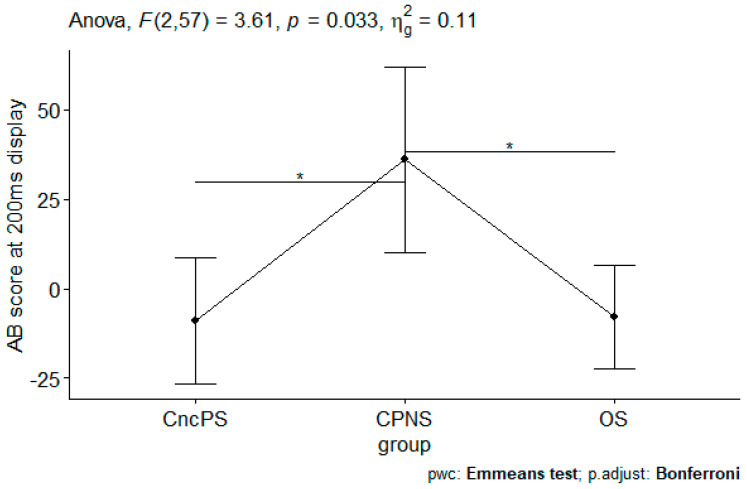
AB scores at 200 ms display when controlling for daily cigarette consumption. CncPS indicates chronic noncancer pain smokers. CPNS indicates chronic pain nonsmokers. OS indicates smokers without pain. AB indicates attentional bias. * *p* < 0.05.

ANCOVA was run to determine group differences (chronic noncancer pain smokers, chronic pain nonsmokers, and smokers without pain) in AB scores at 200 or 2000 ms display after controlling for years of smoking and daily cigarette consumption. No group effect was found when controlling for years of smoking (all *p*s > 0.435). When controlling for daily cigarette consumption, there was a significant group effect on AB scores at 200 ms display (Table 1 and Table 2). Post hoc analysis showed that chronic pain nonsmokers displayed significant AB for opioids when compared to the two other groups (Figure 2). There was no difference in the AB between chronic noncancer pain smokers and smokers without pain. 

#### 3.2.2. Analysis on Reaction Time

**Figure 3:**
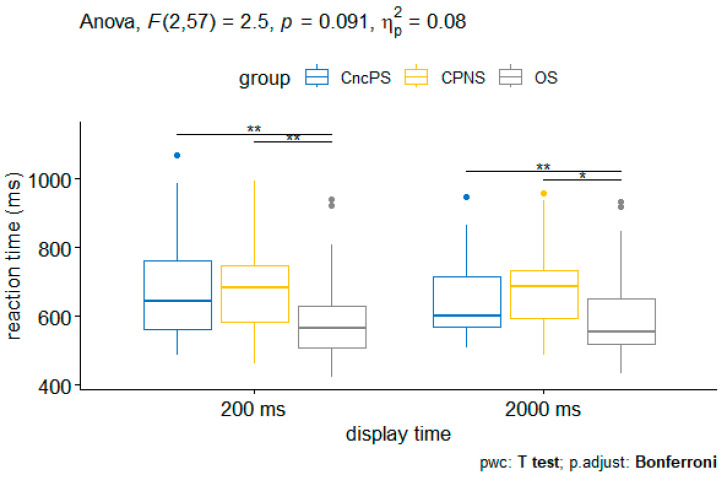
Reaction time of each group when controlling for daily cigarette consumption. CncPS indicates chronic noncancer pain smokers. CPNS indicates chronic pain nonsmokers. OS indicates smokers without pain. * *p* < 0.05, ** *p* < 0.01.

When controlling for daily cigarette consumption, there were significant group effects and significant interaction between group × display time (Table 3). Therefore, the simple main effect of display time on the reaction time of each group was calculated. The simple pairwise comparisons using the Bonferroni method showed that smokers without pain consistently reacted faster in all scenarios compared to the two other groups. No significant difference in reaction time was found between chronic noncancer pain smokers and chronic pain nonsmokers (Figure 3).

## 4. Discussion

Previous studies have warned that smoking may be associated with an increased risk of opioid misuse [2,59]. Using a visual probe task, the current study investigated this topic in the rarely discussed Taiwanese population. We asked the question: How the concomitant use of cigarettes and opioids influences an individual at the attentional level? Parts of the current result agreed with the previous finding: Opioid users displayed significant AB for opioid cues presented for 200 ms but not for cues presented for 2000 ms [24]. Unlike previous studies, with the majority of which were completed in Western countries, the AB for opioids was presented in chronic pain nonsmokers in the current study, rather than the group of interest, the chronic noncancer pain smokers. To paraphrase, smoking habit did not increase the risk of AB for opioids in chronic pain patients who were on long-term opioid treatment. Results on reaction time showed that smokers without pain consistently responded faster than the opioid groups to the task. This could reflect a general psychomotor retardation as a result of long-term opioid therapy [60,61]. 

AB is established through classical conditioning that pairs the substance-related cues with the rewarding effects of the substances [25]. Attention orientation toward the substances and the drug-seeking behaviors could be the resulting response of this conditioning [62]. Attention can be captured and held by the favored substance [63], leading to an elevated risk of misuse [34]. However, some research suggested that the clinical relevance of AB is yet to be determined [25,62,64]. A narrative review showed that most pain patients who were on long-term opioid therapy emphasized the importance of regaining functional status with the help of this medication, despite their feeling of being stigmatized by the clinicians and people around them [65]. Using opioids becomes an acceptable trade-off for a better quality of life. 

One of the potential reasons for the non-significant result found in chronic noncancer pain smokers is that most previous studies included participants who were diagnosed with substance use disorder [62], reported a history of misuse [34], or fitted the diagnosis of opioid dependence [24,33]. However, none of the participants in the current study had received these diagnoses before. Another reason for the non-significant results found in the chronic noncancer pain smokers is that perhaps chronic noncancer pain smokers in the current study did not regard these substances as their target of interest. Although it is beyond the scope of this investigation, scoring over the cutoff 5 on the Fagerström Test for Nicotine Dependence might indicate that these participants were dependent on cigarettes. Could cigarettes be the target of interest? On the other hand, for chronic pain nonsmokers, opioids might be the only hope for improvements in the quality of life [59]. As found in a previous study, AB for a shortly displayed opioid-related picture was correlated to the degree of pain relief in non-dependent users [24]. The expectation of pain relief could lead to the quick orientation to related cues when present. 

There is contextual base for the fear of opioids [66,67]. Among the many factors influencing the public’s attitudes toward prescription opioids (for controlling noncancer pain), race and physician training were specifically pointed out by different studies [66,68,69]. In the US, the increase in overdose and death rates related to opioids has caught public attention [67,70]. As part of the efforts to address this so-called opioid crisis, public health bureaus have released guidelines to regulate the prescription of opioids [71,72]. In contrast to the situation in the US, many countries in Asia put emphasis on the accessibility to prescription opioids [39]. Formulary availability and complex regulation of the prescription of opioid analgesics limit the prescription of these medications [40]. The use of prescription opioids seemed to concern clinicians more than it bothers the patients treated with opioids. A survey in Taiwan showed that most physicians monitor very closely the use of opioids due to their concerns about the pharmacological side effects and socioeconomic problems such as addiction [68]. Rather, from a patient’s perspective, most patients in a South Korean sample who have been prescribed opioids tended to express neutral or positive perspectives toward opioids [73]. These contextual differences might make a difference in how patients from different backgrounds perceive this medicine. This explains for the inconsistencies in attentional presentation between this study and previous studies. 

Although the present study did not find an increased risk of AB for opioids in chronic noncancer pain smokers, this does not imply that smoking does no harm to individuals with pain. Smoking may be adapted to cope with the anxiety associated with the original source of pain or with withdrawal pain [8,74]. Patients may use the drug due to the hypersensitization experienced during the wearing-off phase of the medication [75]. The cross-tolerance between nicotine and opioids can still be the risk factor for escalated use of cigarettes and prescription opioids, resulting in a higher possibility of side effects. 

One limitation of this study is the relatively small sample size for the chronic noncancer pain smokers and chronic pain nonsmokers groups. Unequal cell sizes might have compromised the power of analysis. In an ANCOVA with unbalanced subgroup frequencies, there may be a problem with a loss of power regarding the retaining of unnecessary interaction terms. This would lead to a less adequate or misleading model [76]. The difficulty in recruiting participants with chronic pain who were receiving opioids for pain management is due to the policy that governs this. Patients prescribed opioids are registered in the Taiwan FDA system for strict monitoring and are required to visit the clinic at least once every 14 days. Although frequent monitoring ensures patient safety, it indirectly limits the population of users.

## 5. Conclusions

This study’s results did not support the notion that smoking increases the use of prescription opioids nor did the current results indicate a higher risk of AB for opioids in pain smokers, even after accounting for daily cigarette consumption. Formulary availability and regulatory limitations might have affected patient’s perception of prescription opioids in Taiwan. However, parts of the findings agreed with previous research that some opioid users (for pain control) showed a quick orientation of attention to opioid-related pictures. From previous research, this AB for related cues was correlated with the expectation of pain relief [24]. Results from the analysis on reaction time also agreed that long-term use of opioids might be associated with general psychomotor retardation. A future direction of study would be to examine how chronic noncancer pain smokers regard cigarette-related cues and opioid-related cues. Even though the physiological mechanism of cross-tolerance between the two substances has been studied [69], there have not been direct comparisons between the attentional processing of the two substances in concurrent users. 

## Figures and Tables

**Table 1:** Demographics (*N =* 61).

	Pain Smokers (*n* = 17)		Pain Nonsmokers (*n* = 16)		Smokers without Pain (*n* = 28)		*F*(2, 58)	*p*
	Mean	(*SD*)	Mean	(*SD*)	Mean	(*SD*)		
Daily cigarette consumption	20.88	(7.12)	0	0	21.07	(8.32)	*t* = −0.08	0.936
Years of smoking	28.24	(6.86)	1.44	(5.01)	24.22	(10.28)	*t* = 1.75	0.076
FTND ^a^	6.65	(2.23)			6.11	(1.37)	*t* = 0.90	0.378
Age	47.06	(7.71)	46.44	(8.10)	41.18	(10.82)	2.70	0.075
Education	11.82	(2.63)	13.75	(2.05)	12.11	(2.42)	3.23	0.047
CESD ^b^	25.59	(12.63)	24.50	(10.80)	10.89	(7.39)	15.32	***
BAI ^c^	14.29	(10.78)	16.75	(8.87)	4.64	(4.72)	14.59	***
Pain scores	5.35	(1.21)	5.36	(1.57)	0.96	(1.42)	74.10	***
MME ^d^	253.71	(132.24)	208.61	(178.17)			*t* = 0.82	0.418
AB scores ^e^							*F*(2, 57)	
200 ms	−9.03	(8.75)	36.09	(12.92)	−7.94	(7.21)	3.61	0.033
2000 ms	−1.07	(9.09)	6.24	(13.42)	8.65	(7.49)	0.43	0.656

Footnote: This table displays the analysis of covariance on AB scores when controlling for daily cigarette consumption. ^a^ FTND indicates the Fagerström Test for Nicotine Dependence. ^b^ CESD indicates Center for Epidemiologic Studies Depression Scale. ^c^ BAI indicates the Beck Anxiety Inventory. ^d^ MME indicates Morphine Milligram Equivalent. ^e^ AB scores indicate attentional bias scores. *** *p* < 0.001.

**Table 2:** Statistics of mean reaction time under different conditions and AB scores after controlling for daily cigarette consumption.

	Display Time		AB Scores for Opioid	
	200 ms	2000 ms	200 ms	2000 ms
CncPs ^a^			−9.03 (8.75)	−1.07 (9.09)
Incongruent	675.88 (155.12)	652.40 (124.12)		
Congruent	678.68 (142.00)	652.99 (110.85)		
CPNS ^b^			36.09 (12.92)	6.24 (13.42)
Incongruent	693.24 (120.82)	680.40 (119.05)		
Congruent	675.05 (136.40)	675.54 (113.51)		
OS ^c^			−7.94 (7.21)	8.65 (7.49)
Incongruent	582.51 (116.61)	593.63 (121.95)		
Congruent	584.00 (112.90)	584.48 (116.01)		

Values are displayed as mean (standard error). ^a^ CncPS indicates chronic noncancer pain smokers. ^b^ CPNS indicates chronic pain nonsmokers. ^c^ OS indicates smokers without pain.

**Table 3:** Summary of Analysis of Covariance on reaction times when controlling for daily cigarette consumption.

	Source of Variance	*F*	*df*	*p*	*η_p_* ^2^
Main effects					
	Daily cigarette (co-variate)	2.75	(1, 57)	0.102	0.046
	Group	5.31	(2, 57)	0.008	0.157
	Display time	2.15	(1, 57)	0.148	0.036
	Probe location	0.26	(1, 57)	0.612	0.005
Interactions					
	Daily cigarette × Probe location	1.42	(1, 57)	0.239	0.024
	Display time × Group	3.39	(2, 57)	0.041	0.106
	Probe location × Group	1.66	(2, 57)	0.200	0.055
	Daily cigarette × Display time	0.71	(1, 57)	0.402	0.012
	Display time × Probe location	1.47	(1, 57)	0.231	0.025
	Daily cigarette × Probe location × Display time	1.85	(1, 57)	0.179	0.023
	Display time × Probe location × Group	2.50	(2, 57)	0.091	0.081

## Data Availability

Raw data can be obtained upon request to the corresponding author.

## Supplementary Materials

The following supporting information can be downloaded at https://www.mdpi.com/article/10.3390/medicina60071107/s1, R analytic codes for the visual probe task.
